# Fecal miR-146a as a Non-Invasive Biomarker for *Helicobacter pylori*-Associated Gastritis

**DOI:** 10.3390/life16050759

**Published:** 2026-05-01

**Authors:** Olga Brusnic, Adrian Boicean, Samuel Bogdan Todor, Paula Anderco, Cristian Ichim

**Affiliations:** 1 Department of Internal Medicine VII, George Emil Palade University of Medicine, Pharmacy, Science and Technology of Targu Mures, Gheorghe Marinescu Street No. 38, 540136 Targu Mures, Romania; brusnic_olga@yahoo.com; 2 Faculty of Medicine, Lucian Blaga University of Sibiu, 550169 Sibiu, Romania; samuelbogdant@gmail.com (S.B.T.); cristian.ichim@ulbsibiu.ro (C.I.)

**Keywords:** *Helicobacter pylori*, miR-146a, gastritis, inflammation, biomarker

## Abstract

Background: *Helicobacter pylori* remains a major cause of chronic active gastritis and a clinically relevant precursor of peptic ulcer disease and gastric neoplasia. Host-derived non-invasive biomarkers that reflect infection-related gastric inflammation are still insufficiently developed. This study evaluated the clinical relevance of fecal miR-146a in patients with *H. pylori*-associated gastritis. Methods: We conducted a prospective study over a 3-year period (2023–2025) at the County Clinical Emergency Hospital Sibiu, Romania. The study included 85 adults: 45 patients with confirmed *H. pylori*-associated gastritis and 40 controls. Demographic, clinical, inflammatory, endoscopic, histopathological, and molecular data were analyzed. Continuous variables were compared using the Mann–Whitney U test and categorical variables using the chi-square or Fisher’s exact test. Multivariable analysis was performed using Firth’s penalized logistic regression. Results: Patients with *H. pylori*-associated gastritis showed significantly higher fecal miR-146a expression than controls (2.05 [1.77–2.37] vs. 0.88 [0.77–0.99], *p* < 0.001). They also had higher CRP, ESR, WBC, abdominal pain scores, and a greater burden of endoscopic and histopathological abnormalities. In both multivariable models, fecal miR-146a remained the only significant variable associated with disease status. Conclusions: Fecal miR-146a is markedly elevated in *H. pylori*-associated gastritis and may represent a promising non-invasive biomarker of infection-related gastric inflammation. Larger prospective studies are needed for validation.

## 1. Introduction

*Helicobacter pylori* remains one of the most common chronic bacterial infections worldwide, with a recent systematic review and meta-analysis estimating that 43.1% of the global population was infected during the 2011–2022 period despite a gradual secular decline in prevalence [1]. Its clinical importance derives not only from its frequency but also from its remarkable capacity to colonize the gastric epithelium for decades and to persist lifelong in the absence of eradication therapy [2]. This biological persistence underpins the current concept that *H. pylori* gastritis should be regarded as an infectious disease, even before overt ulceration or neoplastic transformation becomes evident [3].

From a pathophysiological perspective, *H. pylori*-related gastric disease spans a continuum from chronic inflammation to atrophy, intestinal metaplasia, dysplasia, and ultimately gastric carcinogenesis [4]. For this reason, early recognition of infection-related gastritis has value beyond symptom control, as eradication constitutes a key strategy for primary prevention of gastric cancer and for the management of precancerous gastric conditions [5].

Current diagnostic algorithms rely on a combination of invasive methods, including histology, culture, and rapid urease testing on biopsy material, and non-invasive methods such as the urea breath test and stool antigen assays [6]. The urea breath test is a well-established, non-invasive, and clinically useful method for detecting active *H. pylori* infection. However, pathogen-directed tests primarily determine whether an active infection is present and do not directly quantify the intensity of the host inflammatory response or the histological severity of gastritis. Therefore, host-derived molecular biomarkers should be regarded as potentially complementary tools rather than replacements for established diagnostic methods [7].

Stool antigen testing, while clinically valuable, remains susceptible to both preanalytical and analytical sources of variability, including factors related to sample handling, antigen stability, and fluctuations in bacterial load, which may consequently affect the reliability and interpretation of results in routine clinical practice [8]. These limitations have increased interest in host-derived molecular biomarkers that might reflect not only the presence of the microorganism but also the inflammatory response it elicits within the gastric mucosa [9].

Among such candidates, microRNAs are particularly attractive because they function as post-transcriptional regulators of immune and epithelial signaling and are measurably altered in *H. pylori*-infected gastric tissue [10]. miR-146a is of special interest in this setting, as experimental work has shown that *H. pylori* upregulates miR-146a in gastric epithelial cells and mucosal tissue through NF-kB-dependent pathways, while miR-146a in turn modulates IRAK1- and TRAF6-related inflammatory signaling [11].

Human studies have further supported its biological relevance by demonstrating increased miR-146a expression in gastric lesions associated with *H. pylori* infection across different age groups and inflammatory phenotypes [12]. At the same time, fecal microRNAs have emerged as promising non-invasive analytes because they can be detected in stool and exhibit relative stability, preservation, and abundance within this complex biological matrix [13].

This matrix is especially appealing for biomarker development in gastrointestinal disease because stool provides a more localized representation of luminal and mucosal processes than systemic biospecimens such as blood [14]. Indeed, recent studies have shown that fecal microRNA signatures can discriminate gastrointestinal pathology with clinically meaningful accuracy, particularly in colorectal neoplasia, supporting the broader translational potential of stool-based RNA profiling [15].

By contrast, the development of host-derived non-invasive biomarkers for *H. pylori*-associated gastric disease remains comparatively immature, and existing reviews still emphasize the need for more robust transcriptomic and inflammatory marker validation before clinical implementation [16,17]. The rationale for such biomarkers does not arise from a failure of current tests to detect active infection but from the fact that pathogen-directed assays provide limited information regarding the host inflammatory phenotype, histological activity, or biological heterogeneity of gastritis. Within this framework, investigating fecal miR-146a in patients with confirmed *H. pylori*-associated gastritis is biologically plausible and clinically relevant, as it links a well-characterized immune-regulatory microRNA to an accessible non-invasive sample type and may provide complementary information on infection-related gastric inflammation [18].

Accordingly, the present study was designed to compare the clinical, biochemical, endoscopic, histological, and molecular profiles of patients with *H. pylori*-associated gastritis and controls, with particular emphasis on the potential value of fecal miR-146a as a marker of infection-related gastric inflammation.

## 2. Materials and Methods 

### 2.1. Study Design and Clinical Data Collection

This prospective study was conducted over a three-year period, from January 2023 to December 2025, at the County Clinical Emergency Hospital Sibiu, Romania. The aim of the study was to evaluate the clinical, biochemical, endoscopic, histopathological, and molecular differences between patients with confirmed *Helicobacter pylori*-associated gastritis and controls, with particular emphasis on the potential role of fecal miR-146a as a non-invasive biomarker of infection-related gastric inflammation. The final study population comprised 85 participants, including 45 patients with confirmed *H. pylori*-associated gastritis and 40 control subjects.

Participants were assigned to one of two study groups. The case group included patients with confirmed *H. pylori*-associated gastritis. The control group included subjects without confirmed *H. pylori*-associated gastritis, based on the available clinical, endoscopic, and histopathological data. The inclusion and exclusion criteria are presented in Table 1.

### 2.2. Endoscopic and Histopathological Evaluation

The diagnosis of *H. pylori*-associated gastritis was based on histopathological examination of gastric biopsy specimens. Patients were classified as having *H. pylori*-associated gastritis when biopsy specimens showed chronic active gastritis together with histological identification of *H. pylori* organisms. Conversely, control subjects were defined as participants without histological evidence of *H. pylori* organisms in gastric biopsy specimens and without histopathological criteria for *H. pylori*-associated gastritis. Histopathological assessment was performed according to the Updated Sydney System, and biopsy findings were categorized as normal, mild chronic active gastritis, moderate chronic active gastritis, or severe chronic active gastritis. During upper gastrointestinal endoscopy, gastric biopsy specimens were obtained according to routine clinical practice from the antrum and corpus, with additional sampling from the incisura angularis when considered necessary by the endoscopist. Most control subjects had normal gastric biopsy findings; however, three controls showed mild chronic active gastritis without histological identification of *H. pylori* organisms. These participants were retained in the control group because they did not fulfill the predefined criteria for *H. pylori*-associated gastritis. Clinical, demographic, laboratory, endoscopic, histopathological, and molecular data were prospectively recorded and completed, where necessary, from the corresponding medical records. The following variables were recorded for each participant: age, sex, residential setting, body weight, height, body mass index (BMI), Charlson comorbidity index, abdominal pain score, prior antibiotic use within the previous 3 months, proton pump inhibitor (PPI) use in the previous 4 weeks, and family history of *H. pylori* infection or peptic ulcer disease. The inflammatory biomarkers included in the analysis were CRP, ESR, and WBC count. These parameters were analyzed as continuous variables and compared between the two study groups. Serum C-reactive protein levels were interpreted according to the institutional reference range (0–5.0 mg/L).

Abdominal pain severity was assessed using a 0–10 score, with higher values indicating greater symptom intensity. These variables were selected in order to characterize the study population and to explore their relationship with *H. pylori*-associated gastritis.

All included participants underwent upper gastrointestinal endoscopy as part of their routine diagnostic evaluation. Endoscopic reports were systematically reviewed, and findings involving the esophagus, stomach, and duodenum were recorded for analysis. Esophageal findings were categorized as either normal or consistent with esophagitis, while duodenal findings were classified as normal or indicative of duodenitis. Gastric mucosal appearance was assessed endoscopically and categorized as normal or hyperemic.

The primary outcome of the study was the presence of confirmed *H. pylori*-associated gastritis. The main molecular exposure of interest was fecal miR-146a relative expression. Secondary variables included demographic, anthropometric, clinical, inflammatory, endoscopic, and histopathological characteristics.

### 2.3. Stool Sample Processing and miRNA Quantification

Stool samples were collected from all participants at the time of clinical evaluation and processed as soon as possible after collection. Aliquots of fecal material were transferred into sterile microtubes, homogenized in TRIzol reagent (ThermoFisher Scientific, Waltham, MA, USA) according to the manufacturer’s instructions, and stored at −80 °C until further analysis. All samples were handled under conditions minimizing RNA degradation and processed in a standardized manner.

Total RNA, including microRNA, was extracted using TRIzol reagent and resuspended in RNase-free water. miRNA quantity and quality were assessed using fluorometric methods. Fecal miR-146a expression was quantified by reverse transcription quantitative PCR (RT-qPCR) using TaqMan Advanced miRNA Assays (ThermoFisher Scientific), according to the manufacturer’s protocol, and normalized to miR-26c. cDNA synthesis and amplification steps were performed under standardized conditions, and all reactions were run in triplicate.

Relative miR-146a expression levels were calculated using the 2^−ΔCt^ method, normalized to an endogenous reference control. The resulting expression values were treated as continuous variables and used for subsequent statistical analyses.

### 2.4. Statistical Analysis

All statistical analyses were performed using Python (version 3.12; Python Software Foundation, Wilmington, DE, USA) in PyCharm (version 2023.2.1; JetBrains s.r.o., Prague, Czech Republic). Continuous variables are expressed as median and interquartile range (IQR), while categorical variables are reported as frequency and percentage. Between-group comparisons were performed using the Mann–Whitney U test for continuous variables and the chi-square test or Fisher’s exact test for categorical variables, as appropriate. A two-tailed *p*-value < 0.05 was considered statistically significant. The distribution of fecal miR-146a expression between groups was visualized using a raincloud plot combining individual data points, a half-violin density estimate, and a box-and-whisker plot.

Given the modest sample size (*N* = 85) and the potential for quasi-complete separation introduced by fecal miR-146a, multivariable analysis was performed using Firth penalized logistic regression in order to reduce small-sample bias and improve model stability. Continuous predictors included in the multivariable models were standardized (z-scored) prior to model fitting; therefore, odds ratios were expressed per one standard deviation increase. Two multivariable models were examined: Model 1 included miR-146a, age, BMI, and abdominal pain score, while Model 2 included miR-146a, BMI, CRP, and PPI use.

### 2.5. Ethical Considerations

The study was conducted in accordance with the principles of the Declaration of Helsinki and with institutional ethical standards. The analysis used anonymized clinical, laboratory, endoscopic, histopathological, and molecular data, and no information allowing patient identification was included in the study database or manuscript. No intervention beyond routine diagnostic care was performed for the purpose of this analysis, and no additional risk was imposed on the participants. Patient data were handled confidentially and analyzed in anonymized form, in accordance with local regulations and institutional requirements.

## 3. Results

In this study, we analyzed 85 patients (40 controls and 45 with confirmed *H. pylori*-associated gastritis) to investigate the clinical, biochemical, and molecular features distinguishing the two groups, as well as the independent predictive value of fecal miR-146a expression. Baseline demographic and clinical characteristics are summarized in Table 2. Multivariable associations were subsequently assessed using Firth’s penalized logistic regression, owing to the modest sample size and the potential for quasi-complete separation introduced by the strong discriminatory capacity of miR-146a.

### 3.1. Baseline Demographic and Clinical Characteristics

The two groups were broadly comparable in terms of age (controls: median 50.00 years, IQR 34.00–62.00; *H. pylori*: 44.00 years, IQR 27.00–64.00; *p* = 0.283), sex distribution (50.0% female in each group; *p* = 0.919), and residential setting (*p* = 0.611). However, patients with *H. pylori* gastritis had significantly higher body weight (70.40 vs. 53.85 kg; *p* = 0.005) and BMI (21.40 vs. 18.85 kg/m^2^; *p* < 0.001).

Inflammatory markers were markedly elevated in the *H. pylori* group: CRP (5.19 vs. 1.29 mg/L; *p* < 0.001), ESR (21.80 vs. 9.00 mm/h; *p* < 0.001), and WBC (9.01 vs. 6.96 × 10^9^/L; *p* < 0.001). Abdominal pain scores were substantially higher in *H. pylori*-positive patients (median 6.00 vs. 2.00; *p* < 0.001). Fecal microRNA expression was strongly elevated in the infection group: miR-146a (2.05 vs. 0.88; *p* < 0.001).

Endoscopic and histological findings differed significantly between the groups. Esophagitis was more prevalent in the *H. pylori* cohort (28.9% vs. 10.0%; *p* = 0.030), as was duodenitis (31.1% vs. 7.5%; *p* = 0.007). Polymorphonuclear (PMN) infiltration, a hallmark of active infection, was present in 95.6% of *H. pylori*-positive patients versus only 17.5% of controls (*p* < 0.001). Biopsy-confirmed chronic active gastritis was found in all 45 infected patients (42.2% mild, 40.0% moderate, 17.8% severe), compared with only 3 controls (7.5% mild; *p* < 0.001). PPI use in the preceding four weeks was significantly more common in the *H. pylori* group (37.8% vs. 5.0%; *p* < 0.001). Because only three control participants showed mild chronic active gastritis, this subgroup was considered too small for separate graphical or statistical analysis. Therefore, these cases were retained within the control group, and their potential relevance for false-positive fecal miR-146a findings should be specifically addressed in larger validation cohorts.

Fecal miR-146a relative expression was markedly elevated in the *H. pylori* gastritis group compared to controls (median 2.05, IQR 1.77–2.37 vs. 0.88, IQR 0.77–0.99; *p* < 0.001). This difference was among the most statistically robust findings in the entire table, alongside inflammatory markers and histological findings. Notably, the IQRs of the two groups showed virtually no overlap; the lower bound of the *H. pylori* group (1.77) exceeded the upper bound of the control group (0.99), indicating a near-complete separation in expression values between the two cohorts, which underlies the strong discriminatory capacity of this biomarker observed in subsequent multivariable analyses.

### 3.2. Multivariable Analysis: Firth’s Penalized Logistic Regression

Given the binary outcome (*H. pylori* gastritis vs. control) and the small-to-moderate sample size (N = 85), Firth’s penalized logistic regression was applied for all multivariable models to minimize small-sample bias and address potential quasi-complete separation. All continuous predictors were standardized (z-scored) prior to analysis; odds ratios (ORs) are therefore expressed per one standard deviation (SD) change.

Model 1: miR-146a + Age + BMI + Abdominal Pain Score

The first model included fecal miR-146a expression, age, BMI, and abdominal pain score as covariates (Table 3). miR-146a was the sole statistically significant predictor (OR per 1 SD = 2069.207; 95% CI: 7.741–553,102.488; *p* = 0.007), reflecting an extraordinarily strong association between elevated fecal miR-146a and *H. pylori* gastritis status. Age (OR = 0.668; 95% CI: 0.141–3.160; *p* = 0.611), BMI (OR = 0.781; 95% CI: 0.174–3.511; *p* = 0.747), and abdominal pain score (OR = 1.680; 95% CI: 0.230–12.253; *p* = 0.609) did not reach statistical significance after adjustment. The wide confidence intervals for miR-146a are consistent with the large effect size and limited sample size, but statistical significance was clearly maintained.

Model 2: miR-146a + BMI + CRP + PPI Use

The second model examined miR-146a alongside BMI, CRP, and PPI use as additional clinically relevant covariates (Table 4). miR-146a again emerged as the only statistically significant predictor (OR per 1 SD = 445.761; 95% CI: 10.839–18,331.909; *p* = 0.001). PPI use showed a numerically elevated OR (9.024; 95% CI: 0.189–430.849; *p* = 0.265), consistent with its higher prevalence in the *H. pylori* group on univariate analysis, though this did not reach significance in the multivariable model. BMI (OR = 1.489; *p* = 0.579) and CRP (OR = 1.557; *p* = 0.694) were similarly non-significant, suggesting that miR-146a captures the disease signal independently of these inflammatory and metabolic markers.

### 3.3. Distribution of Fecal miR-146a Expression: Controls vs. H. pylori Positive

**Figure 1:**
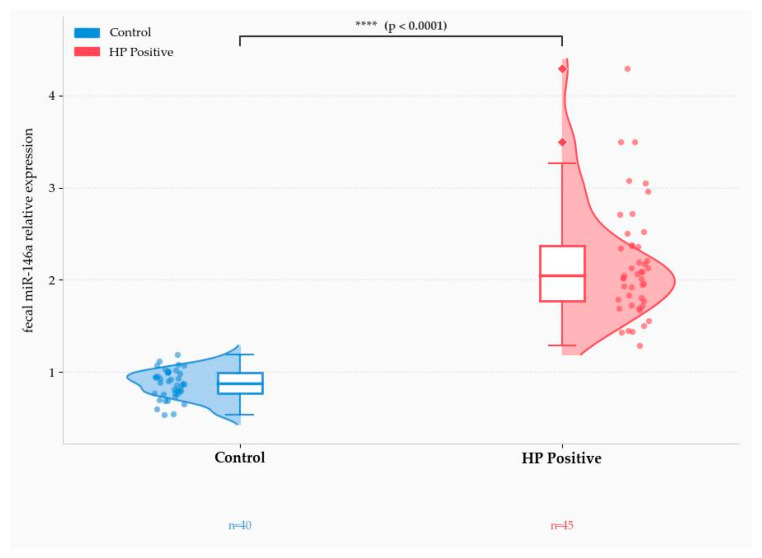
miR-146a: Controls vs. HP Positive. Raincloud plot of fecal miR-146a relative expression in control (*n* = 40) and H. pylori-positive (HP-positive; *n* = 45) groups.

Fecal miR-146a relative expression was compared between the control group (*n* = 40) and the *H. pylori*-positive (HP-positive) group (*n* = 45) using the two-sided Mann–Whitney U test. As illustrated in Figure 1, miR-146a expression levels were markedly higher in HP-positive individuals compared to controls, with the difference reaching statistical significance (*p* < 0.0001). The raincloud plot reveals a clear rightward shift in the distribution of miR-146a expression values among HP-positive patients, as evidenced by both the density estimate and the individual data points. The median expression level in the HP-positive group was substantially greater than that observed in the control group, with minimal overlap between the interquartile ranges of the two distributions. Notable heterogeneity in miR-146a expression was observed within the HP-positive group, as reflected by the wider density spread and the presence of high-expression outliers. This heterogeneity should be interpreted in relation to the histological spectrum of the HP-positive group, which included 19 patients with mild chronic active gastritis, 18 with moderate chronic active gastritis, and 8 with severe chronic active gastritis. Although the present sample size does not allow a robust severity-stratified analysis, these findings support the need for future studies specifically designed to determine whether fecal miR-146a varies according to histological activity. Taken together, these findings indicate that fecal miR-146a is differentially expressed in the context of *H. pylori* infection and may serve as a candidate non-invasive biomarker for its detection (Figure 1).

## 4. Discussion

The present study demonstrates that patients with *Helicobacter pylori*-associated gastritis exhibit a distinct clinical and biological profile characterized by higher fecal miR-146a expression, greater systemic inflammatory activation, and more pronounced endoscopic and histopathological abnormalities than controls. Taken together, these findings support the view that *H. pylori*-associated gastritis is not merely a state of bacterial colonization but a biologically active inflammatory condition in which host-response signals can be detected beyond tissue biopsy. This perspective is consistent with current models of *H. pylori* pathogenesis, which emphasize the interplay between persistent gastric colonization, mucosal immune activation, epithelial injury, and progressive structural remodeling of the gastric mucosa [19,20].

Within this multidimensional disease framework, fecal miR-146a appears to be the most informative molecular signal identified in our cohort. Its strong between-group separation and retained association with disease status after multivariable adjustment are biologically plausible, given the established role of miR-146a in *H. pylori*-related inflammatory signaling. Recent molecular and translational work has reinforced the importance of microRNA dysregulation in the evolution of *H. pylori*-related gastric disease, with miR-146a repeatedly implicated in inflammatory and epithelial regulatory networks linked to chronic infection. Clinical evidence has also continued to accumulate in support of miR-146a as a relevant marker in *H. pylori*-associated gastritis, including recent pediatric data suggesting discriminatory potential in infected patients [4,18]. The heterogeneity of fecal miR-146a expression within the *H. pylori*-positive group should not be attributed exclusively to host-related variability. Differences among *H. pylori* strains may also contribute to variation in inflammatory activity and molecular response. Virulence determinants such as CagA, VacA, OipA, and other strain-specific factors have been implicated in the intensity of gastric inflammation and disease phenotype. Because *H. pylori* genotyping was not performed in the present study, we could not determine whether fecal miR-146a expression differed according to bacterial virulence profiles. This limitation has been added to the manuscript and should be addressed in future studies.

A central strength of the present study is the use of stool rather than gastric tissue or blood as the matrix for biomarker assessment. From a translational perspective, fecal microRNAs are attractive because they are non-invasive, biologically proximal to gastrointestinal pathology, and increasingly recognized as plausible indicators of mucosal or luminal disease processes. Contemporary reviews in digestive medicine now place microRNAs among the most promising biomarker classes across gastrointestinal disorders, while broader work on fecal miRNAs suggests that stool-based RNA signals may capture not only epithelial injury but also disease-relevant host–microenvironment interactions. In this context, our data extend the emerging logic of stool RNA biomarker research into *H. pylori*-associated gastritis, a field that remains substantially less developed than colorectal or inflammatory bowel disease biomarker research [21].

The clinical relevance of this approach should be interpreted within a complementary rather than a substitutive diagnostic framework. Non-invasive tests such as the urea breath test and stool antigen testing remain central to the routine detection of active *H. pylori* infection. However, these tests primarily establish infection status and do not directly characterize the intensity of the host inflammatory response or the histological activity of gastritis. Conversely, biopsy-based methods provide histological information but are invasive and may be influenced by sampling strategy, medication exposure, and lesion distribution. Therefore, fecal miR-146a may be most relevant as a non-invasive host-response marker that could enrich disease characterization, particularly if future studies confirm associations with histological severity, inflammatory activity, or clinically relevant disease phenotypes [22,23].

The inflammatory profile observed in our cohort further supports the internal coherence of the findings. Patients with *H. pylori* gastritis showed higher CRP, ESR, and WBC values, which is directionally consistent with contemporary evidence linking *H. pylori* infection to systemic and mucosal inflammatory activation. Recent work has continued to associate *H. pylori*-related gastritis with elevated hs-CRP and with broader cytokine-driven immune responses, including IL-17-related signaling and other pathways involved in chronic gastric inflammation and progression to more advanced pathology. Accordingly, the coexistence of elevated fecal miR-146a and higher inflammatory markers in our study strengthens the interpretation that miR-146a is capturing a biologically meaningful component of the host inflammatory response, rather than representing an isolated molecular anomaly [24,25].

The endoscopic and histopathological differences between groups are equally important when interpreting the molecular findings. The predominance of gastric hyperemia, polymorphonuclear infiltration, and chronic active gastritis in infected patients corresponds closely to the recognized tissue phenotype of active *H. pylori* disease. Recent histopathology-focused reviews emphasize that neutrophilic epithelial infiltration and chronic active inflammatory change remain defining features of *H. pylori*-associated gastritis and are central to disease classification. This concordance between the molecular, inflammatory, endoscopic, and histological layers is a notable strength of the present study because it supports the biological credibility of fecal miR-146a as a disease-associated signal within a well-characterized pathological context [20,26].

At the same time, the statistical behavior of miR-146a in our multivariable models requires careful interpretation. The very large odds ratios and extremely wide confidence intervals indicate that the biomarker achieved strong separation between groups, but they also show that the magnitude of effect was estimated with limited precision. For that reason, the use of Firth’s penalized logistic regression was methodologically appropriate, as contemporary statistical literature continues to support bias-reduced penalized estimation when logistic models are affected by small sample size, sparse data, or separation. In this setting, the key message is not the exact numerical size of the odds ratio but the persistence of the association after adjustment and the need to avoid overstating precision in a modest-sized exploratory dataset [27,28].

From a translational standpoint, our findings support a cautious but meaningful conclusion: fecal miR-146a should currently be viewed as a promising candidate biomarker of *H. pylori*-related gastric inflammation, not as a stand-alone diagnostic test ready for routine clinical deployment. The introduction of any additional biomarker assay would require evidence of incremental clinical value, reproducibility, assay standardization, and cost-effectiveness. Therefore, future studies should determine whether fecal miR-146a improves inflammatory phenotyping, prediction of histological activity, risk stratification, or post-treatment monitoring beyond established diagnostic approaches such as urea breath testing and stool antigen testing. More broadly, recent biomarker research in gastrointestinal disease suggests that clinically successful non-invasive platforms are likely to emerge from multi-marker or integrated approaches rather than from isolated signals alone. Against this background, fecal miR-146a may be best positioned as part of a broader host-response biomarker framework for non-invasive characterization of *H. pylori*-associated gastric disease [29,30].

A limitation of this study is the inclusion of exclusively patients with non-atrophic, non-metaplastic gastritis, which precludes the assessment of fecal miR-146a across the full spectrum of *H. pylori*-associated gastric pathology, including atrophic gastritis and intestinal metaplasia. Another limitation is that only three control participants showed mild chronic active gastritis. This number was insufficient to estimate the potential false-positive rate of fecal miR-146a in non-*H. pylori*-associated mild gastritis or to support a separate subgroup analysis. Future studies should include larger control subgroups with non-*H. pylori* gastritis in order to determine whether fecal miR-146a is specific for *H. pylori*-associated inflammation or reflects gastric inflammatory activity more broadly. Another limitation is the absence of *H. pylori* strain genotyping. Therefore, the potential influence of CagA, VacA, OipA, and other virulence determinants on fecal miR-146a expression could not be assessed. Another limitation of this study is the potential influence of recent proton pump inhibitor use on *H. pylori* detection, which may have resulted in false-negative findings in a small subset of patients. Nonetheless, the small number of such cases makes a significant impact on the study results unlikely.

## 5. Conclusions

Fecal miR-146a was markedly elevated in patients with *Helicobacter pylori*-associated gastritis and remained significantly associated with disease status after adjustment for selected covariates, supporting its relevance as a marker of host inflammatory activation in *H. pylori*-related gastric disease. Beyond confirming the molecular involvement of miR-146a in the inflammatory response to infection, our findings suggest that stool-based assessment of this microRNA may offer a promising non-invasive approach for the characterization of infection-related gastric inflammation.

Despite their promise, these findings warrant cautious interpretation given the single-center design and modest sample size. Robust validation in larger, multicenter prospective cohorts, alongside formal evaluation of diagnostic performance metrics, is essential before fecal miR-146a can be translated into clinical practice.

## Figures and Tables

**Table 1:** Inclusion and exclusion criteria of the study population.

Inclusion Criteria	Exclusion Criteria
Age ≥ 18 years	Age below 18 years
Availability of a stool sample for fecal miR-146a analysis	Incomplete clinical, laboratory, endoscopic, histological, or molecular data
Availability of complete demographic and clinical data	Absence of gastric biopsy or unavailable pathology report
Availability of laboratory inflammatory markers, including C-reactive protein (CRP), erythrocyte sedimentation rate (ESR), and white blood cell count (WBC)	Severe concomitant inflammatory, infectious, autoimmune, or neoplastic disorders that could significantly influence systemic inflammation or fecal microRNA expression independently of *H. pylori* infection
Availability of histopathological assessment of gastric biopsy specimens	Previously diagnosed gastric malignancy
	History of gastric surgery
	Lack of an adequate stool sample for molecular analysis

**Table 2:** Baseline demographic, clinical, biochemical, endoscopic, and histological characteristics of the study population (*N* = 85).

Variable	Control (*n* = 40)	*H. pylori* Gastritis (*n* = 45)	*p*-Value
Age (years)	50.00 (34.00–62.00)	44.00 (27.00–64.00)	0.283
Weight (kg)	53.85 (49.08–66.45)	70.40 (52.90–78.10)	**0.005**
Height (cm)	171.40 (160.15–182.00)	180.30 (162.20–195.00)	0.112
BMI (kg/m^2^)	18.85 (17.66–20.54)	21.40 (19.63–22.21)	**<0.001**
Fecal miR-146a (rel. exp.)	0.88 (0.77–0.99)	2.05 (1.77–2.37)	**<0.001**
CRP (mg/L)	1.29 (0.92–2.50)	5.19 (2.71–8.26)	**<0.001**
ESR (mm/h)	9.00 (6.47–11.53)	21.80 (13.90–25.30)	**<0.001**
WBC (×10^9^/L)	6.96 (6.33–7.80)	9.01 (7.81–9.92)	**<0.001**
Charlson Index	0.00 (0.00–0.00)	0.00 (0.00–1.00)	0.129
Abdominal Pain Score (0–10)	2.00 (1.00–3.00)	6.00 (3.00–8.00)	**<0.001**
Sex			
Female	20 (50.0%)	22 (48.9%)	
Male	20 (50.0%)	23 (51.1%)	0.919
Residence			
Rural	13 (32.5%)	17 (37.8%)	
Urban	27 (67.5%)	28 (62.2%)	0.611
Endoscopy—Esophagus			
Esophagitis	4 (10.0%)	13 (28.9%)	
Normal	36 (90.0%)	32 (71.1%)	**0.030**
Endoscopy—Duodenum			
Duodenitis	3 (7.5%)	14 (31.1%)	
Normal	37 (92.5%)	31 (68.9%)	**0.007**
Polymorphonuclear Infiltration (PMN)			
Absent	33 (82.5%)	2 (4.4%)	
Present	7 (17.5%)	43 (95.6%)	**<0.001**
Prior Antibiotic Use (3 months)			
No	28 (70.0%)	24 (53.3%)	
Yes	12 (30.0%)	21 (46.7%)	0.116
PPI Use (last 4 weeks)			
No	38 (95.0%)	28 (62.2%)	
Yes	2 (5.0%)	17 (37.8%)	**<0.001**
Family History of *H. pylori* or Ulcer			
No	31 (77.5%)	27 (60.0%)	
Yes	9 (22.5%)	18 (40.0%)	0.084
Endoscopy—Stomach			
Normal	34 (85.0%)	4 (8.9%)	
Hyperemia	0 (0.0%)	15 (33.3%)	**<0.001**
Biopsy—Stomach Report			
Normal	37 (92.5%)	0 (0.0%)	
Mild chronic active gastritis	3 (7.5%)	19 (42.2%)	
Moderate chronic active gastritis	0 (0.0%)	18 (40.0%)	
Severe chronic active gastritis	0 (0.0%)	8 (17.8%)	**<0.001**

Data are expressed as median (IQR) for continuous variables and n (%) for categorical variables. Comparisons performed using the Mann–Whitney U test (continuous) and chi-square or Fisher’s exact test (categorical). Bold *p*-values indicate statistical significance (*p* < 0.05). IQR = interquartile range; BMI = body mass index; CRP = C-reactive protein; ESR = erythrocyte sedimentation rate; WBC = white blood cell count; PMN = polymorphonuclear infiltration; PPI = proton pump inhibitor; rel. exp. = relative expression.

**Table 3:** Firth’s penalized logistic regression—Model 1: miR-146a + Age + BMI + Abdominal Pain Score (*N* = 85).

Variable	OR (per 1 SD)	95% CI	*p*-Value
miR-146a	**2069.207**	7.741–553,102.488	**0.007**
Age	0.668	0.141–3.160	0.611
BMI	0.781	0.174–3.511	0.747
Abdominal Pain Score	1.680	0.230–12.253	0.609

All continuous variables were standardized (per 1 SD). Bold entries denote statistical significance (*p* < 0.05). OR = odds ratio; CI = confidence interval; BMI = body mass index; SD = standard deviation.

**Table 4:** Firth’s penalized logistic regression—Model 2: miR-146a + BMI + CRP + PPI Use (*N* = 85).

Variable	OR (per 1 SD)	95% CI	*p*-Value
miR-146a	445.761	10.839–18,331.909	**0.001**
BMI	1.489	0.365–6.063	0.579
CRP	1.557	0.171–14.144	0.694
PPI Use	9.024	0.189–430.849	0.265

All continuous variables were standardized (per 1 SD). Bold entries denote statistical significance (*p* < 0.05). OR = odds ratio; CI = confidence interval; BMI = body mass index; CRP = C-reactive protein; PPI = proton pump inhibitor; SD = standard deviation.

## Data Availability

The data presented in this study are available from the corresponding author upon reasonable request due to privacy, legal, and ethical reasons.
